# The Effect of Chronic Laxative Use on Lipid Profile and HbA1c: A Hospital-Based Retrospective Study

**DOI:** 10.7759/cureus.45055

**Published:** 2023-09-11

**Authors:** Ghada Ajabnoor, Basmah Eldakhakhny, Kamal T Hashim, Mohammed M Alzahrani, Rayan Eskandarani, Bader AlQusaibi, Ahmad K Alqarni, Naser M Alsulaimani, Mansour Dahlan, Sumia Enani, Yousef Almoghrabi, Aliaa A Alamoudi, Amani Alhozali, Ayman Elsamanoudy

**Affiliations:** 1 Department of Clinical Biochemistry, Faculty of Medicine, King Abdulaziz University, Jeddah, SAU; 2 Food, Nutrition, and Lifestyle Research Unit, King Fahd Medical Research Centre, King Abdulaziz University, Jeddah, SAU; 3 Department of Clinical Biochemistry, King Abdulaziz University Hospital, Jeddah, SAU; 4 Faculty of Medicine, King Abdulaziz University, Jeddah, SAU; 5 Department of Food and Nutrition, Faculty of Human Sciences and Design, King Abdulaziz University, Jeddah, SAU; 6 Department of Medicine, King Abdulaziz University Hospital, Jeddah, SAU; 7 Department of Medical Biochemistry and Molecular Biology, Faculty of Medicine, Mansoura University, Mansoura, EGY

**Keywords:** kauh, retrospective, hba1c, bmi, hdl-c, tg, ldl-c, cholesterol, chronic laxative

## Abstract

Background: Laxatives are over-the-counter medications used as a treatment for constipation. The lipid-lowering effect of the long-term use of laxatives has been proposed.

Aim: To investigate the possible impact of the chronic use of laxatives on serum lipid profile, body mass index (BMI), and hemoglobin A1c (HbA1c).

Methods: An observational retrospective cohort study was conducted to analyze data related to patients who received laxatives for six or 12 months or more in the KAUH database system. BMI, weight, cholesterol, triglycerides, low-density lipoprotein (LDL), high-density lipoprotein (HDL), aspartate aminotransferase (AST), alanine aminotransferase (ALT), and HbA1c data were collected retrospectively from hospital records for three time points: baseline, six months, and 12 months of laxative treatment from the starting date for each patient.

Results: A total of 106 patients' records fulfilled the inclusion criteria, 46 (43%) males with a mean age of 66 and 60 (57%) females with a mean age of 63. A significant decrease in plasma cholesterol and low-density lipoprotein-cholesterol (LDL-C) levels was observed in those who used laxatives for 12 months. Furthermore, an overall BMI and ALT reduction was seen in the combined. On the other hand, HbA1c levels appeared to improve in the combined group but not statistically significant. The change in the cholesterol level could be observed in patients receiving statin treatment and those without, with no statistical significance between the two groups.

Conclusion: Chronic laxative use for 12 months or more is associated with a decreased total and LDL-C level with no significant effect on high-density lipoprotein-cholesterol (HDL-C) levels. Additionally, there was a significant reduction in BMI and ALT. This effect is more prominent with combined therapy. Further multicentric studies on larger sample sizes are recommended to confirm our findings.

## Introduction

Laxatives are over-the-counter (OTC) medications for acute and/or chronic constipation and other off-label uses [1,2]. Different classes of laxatives are available based on their mechanisms of action, such as bulk forming, osmatic, and stimulant laxatives [3]. Over the years, many studies have indicated the high prevalence of misuse or abuse of laxative medications [4]. Several reasons could lead to the chronic use of laxatives. These include individuals suffering from an eating disorder such as anorexia or bulimia nervosa, middle-aged or older individuals who begin using laxatives when constipated but continue to overuse them, and individuals engaged in certain types of athletic training or sports who use laxatives for weight loss [4]. Other reports have highlighted laxative use for body weight control [4]. However, constipation treatment remains the primary indication of chronic laxative self-medication [5].

The long-term use effect of laxatives on body weight management and lipid metabolism was proposed [6] and reported in a previous study [7]. Moreover, chronic laxative use was reported to be associated with enhanced cholesterol conversion to bile acid and, consequently, lowering the serum level of both cholesterol and low-density lipoprotein-cholesterol (LDL-C) [8]. Polyethylene glycol (PEG) is an osmotic laxative that regulates cholesterol metabolism and modifies plasma levels [9]. In addition, the laxative drug elobixibat (an inhibitor of the ileal bile acid transporter) was associated with reduced LDL-C concentration in Japanese patients with chronic constipation [10,11].

Despite the strong link between obesity and the risk for disease development, various studies have shown the importance of maintaining body mass index (BMI) in chronic diseases. For example, low BMI is a significant chronic obstructive pulmonary disease (COPD) risk factor affecting disease mortality [12,13]. In addition, in a large study of 114,430 patients, a BMI lower than 22.5 was associated with an increased mortality risk compared to a higher BMI in all 23 cancer types [14]. It has been further suggested that, unlike visceral fat, subcutaneous fat can have a protective role in chronic illness through the secretion of protective adipokines and the lowering of proinflammatory markers [15,16]. Such evidence indicates the importance of monitoring and maintaining adequate body weight during chronic diseases. Given that many chronic laxative users are patients with chronic diseases, it is important to evaluate the impact of laxative use on those patients' metabolic profiles and BMI.

To our knowledge, only very limited studies have investigated the effect of laxatives on lipid metabolism and BMI in Saudi Arabia. Furthermore, no published research discussed the potential impact of chronic laxative use on plasma lipid profiles in Saudi Arabia. Therefore, the current study aimed to retrospectively investigate the possible effect of the chronic use of laxatives on the serum lipid profile and HbA1c in Saudi patients who attended King Abdulaziz University Hospital (KAUH).

## Materials and methods

Study design

An observational retrospective cohort study was conducted to analyze data related to inpatients and outpatients who were recorded to be taking laxatives for six months or more in the KAUH database system (Phoenix) approved by the KAUH Research Ethical Committee Ethical Ref No. 192-22.

Inclusion/exclusion criteria 

**Figure 1:**
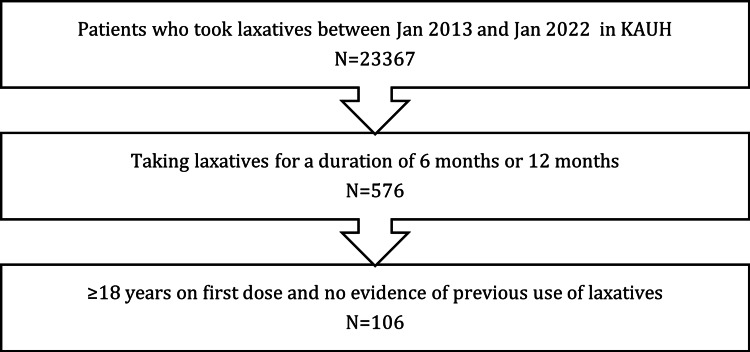
A flow chart presenting the sequence of data collection of patient records according to the inclusion criteria of the study.

The Phoenix database records (Phoenix software) were used to identify adult (≥18 years) patients who had been prescribed laxatives between the 1st of January 2013 and the 1st of January 2022 for six consecutive months or more. Baseline data recording was considered the date before the first dose of laxatives was prescribed. Patients who had at least 50% pre- and post-laxative medication of the following variables: body mass index (BMI), lipid profile, liver function tests (LFT), and Hb1Ac, throughout the determined length of the study were included. Inclusion criteria are summarized in Figure 1. The exclusion criteria included records of patients on chronic use of laxatives six months before the index date or if the laxative dose is less than three days a week. Additionally, excluded data were of patients with familial hypercholesteremia, other genetic dyslipidemia, patients who have undergone bariatric surgery, and those who have taken weight-loss medications as orlistat.

Demographic characteristics and laboratory parameters

The following data were obtained at baseline: age, nationality, gender, height, weight, BMI, history of statin, and any identified comorbidities through electronic data records from the KAUH Phoenix system. Laboratory parameters, including cholesterol, triglycerides (TG), LDL, high-density lipoprotein (HDL), aspartate aminotransferase (AST), alanine aminotransferase (ALT), and HbA1c, were recorded for baseline, six months, and 12 months based on each patient's laxative medication starting date. The very low-density lipoprotein (VLDL) was calculated from the TG using the Friedewald formula [17]. In addition, the type of laxatives, dose, and total duration of consecutive laxative use were recorded. Laxative medications were also categorized based on their mechanism of action: stimulant, bulk-forming, and osmotic. Combined therapy patients are prescribed two different types of laxatives simultaneously.

Statistical analysis

Data were analyzed using IBM SPSS statistics version 26.0 for Windows (IBM Corp, Armonk, NY) and GraphPad Prism version 9.5 (GraphPad Software, San Diego, CA). Data were presented as a mean and standard error of the mean (SEM). The difference between males and females was measured using chi-square. Paired t-test analysis was used to compare the baseline and six months or 12 months. Two-way analysis of variance (ANOVA) was used to compare time points and different laxative groups, followed by one-way ANOVA to study the significant difference between laxative groups, followed by post hoc (Tukey) multivariant analysis for all variables in different groups individually. An independent t-test analysis was used to compare factors between the two groups (statin vs. no statin). P-value <0.05 is considered significant.

## Results

A total of 106 patient records (46 (43%) male and 60 (57%) female) were analyzed. The average age was 64 years. Forty-one patients (39%) were diagnosed with hypertension and 18 with heart failure (17%), while 33 patients had kidney disease (31%). A summary of demographic and anthropometrics can be found in Table 1. Fifty-four patients (51%), 19 (41%) of total males and 35 (48%) of total females, were prescribed a statin. Patients were categorized based on the type of laxatives they used into four groups: osmotic (n = 30, 28.3%), stimulant (n = 33, 31.1%), bulk-forming (n = 16, 15.1%), or combined (n = 27, 25.5%), as shown in Table 2.

**Table 1:** Demographic, anthropometric, and clinical characteristics of studied groups.

	Male n = 46		Female n = 60	
	Mean	SEM	Mean	SEM
Age (years)	66	3	63	2
Height (cm)	165.79	1.27	157.01	0.86
BMI(kg/m^2^)	27.66	0.62	29.89	0.96
	Count	%	Count	%
Nationality				
Saudi	18	39.00%	32	53.00%
Non-Saudi	28	61.00%	28	47.00%
Hypertension				
Yes	17	37.00%	24	40.00%
No	29	63.00%	36	60.00%
Heart failure				
Yes	9	20.00%	9	15.00%
No	37	80.00%	51	85.00%
Liver disease				
Yes	4	9.00%	3	5.00%
No	42	91.00%	57	95.00%
Kidney disease				
Yes	19	41.00%	14	23.00%
No	27	59.00%	46	77.00%
Cancer				
Yes	15	33.00%	12	20.00%
No	31	67.00%	48	80.00%
Chemotherapy				
Yes	6	13.00%	8	13.00%
No	40	87.00%	52	87.00%
Statin				
Yes	19	41.00%	35	58.00%
No	27	59.00%	25	42.00%

**Table 2:** Laxative group based on their mechanism of action.

Laxative mechanism	Count	%
Osmotic	30	28.30%
Stimulant	33	31.10%
Bulk-forming	16	15.10%
Combined	27	25.50%

**Figure 2:**
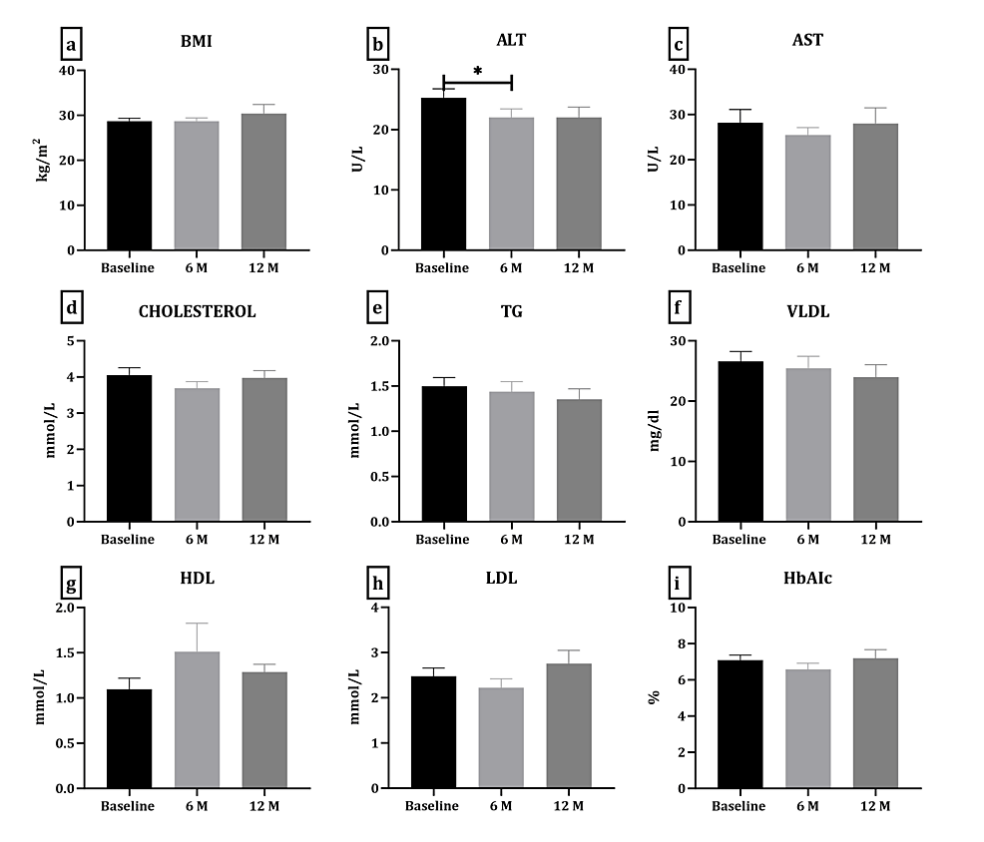
The overall changes in BMI and biochemical parameters in six and 12 months in all patients combined a. BMI, b. ALT, c. AST, d. cholesterol, e. TG, f. VLDL, g. HDL, h. LDL, i. HbA1c. Data presented as mean ± SEM. *p-Value <0.05 calculated by one-way ANOVA followed by post hoc (Tukey) multivariant analysis. BMI, body mass index; ALT, alanine aminotransferase; AST, aspartate aminotransferase; TG, triglycerides; VLDL, very low-density lipoprotein; HbA1c, hemoglobin A1c; ANOVA, analysis of variance.

**Figure 3:**
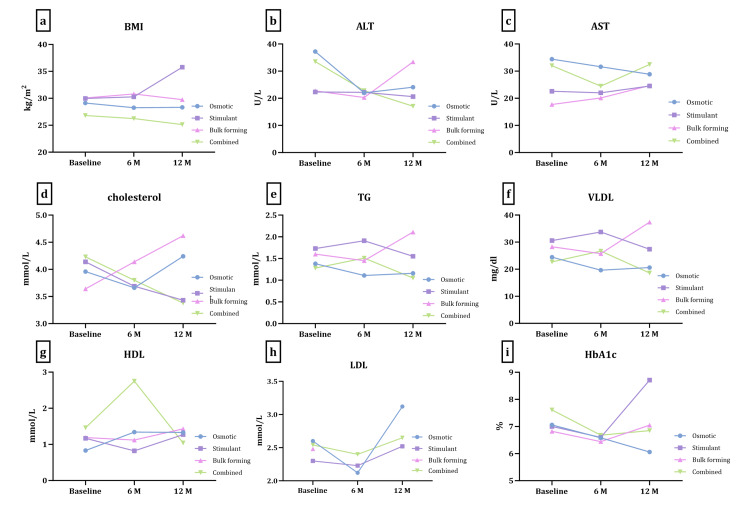
The changes in BMI and biochemical parameters in six and 12 months in different laxative groups a. BMI, b. ALT, c. AST, d. cholesterol, e. TG, f. VLDL, g. HDL, h. LDL, i. HbA1c. Data are presented as the mean of each laxative group. Data were analyzed using two-way ANOVA followed by post hoc multivariant analysis. BMI, body mass index; ALT, alanine aminotransferase; AST, aspartate aminotransferase; TG, triglycerides; VLDL, very low-density lipoprotein; HbA1c, hemoglobin A1c; ANOVA, analysis of variance.

Different parameters and biochemical tests were recorded at baseline (before receiving laxatives) and during follow-up (at six and 12 months). Figure 2 shows the difference in BMI and biochemical parameters at six and 12 months regardless of their laxative use. ALT was significantly changed between baseline and six months (p-value 0.04). Data were further analyzed to examine different laxative groups. A two-way ANOVA was used, looking at the duration and different laxative groups (Figure 3). No statistical difference was observed in any of the parameters in six or 12 months. On the other hand, a significant change was observed in BMI (p-value 0.008), TG (p-value 0.005), VLDL (p-value 0.006), and AST (p-value 0.03) between the different laxative groups.

Due to the small sample number, a paired t-test was performed for patients' parameters in each laxative group to see the difference in six months and 12 months, and one-way ANOVA was followed by post-hoc multivariant analysis to compare these changes between laxative groups. For six months no significance in lipid profile was noted in all four laxative groups. Cholesterol showed a trend toward significance among the combined group, with a decrease from 4.99 (mmol/L) at baseline to 4.08 (mmol/L) at six months with a p-value of 0.06. A trend toward significance was also seen in HbA1c in the combined group, with a reduction in the mean of HbA1c from 7.43% to 6.68% with a p-value of 0.09. There was also a noted change in ALT levels in the osmotic group, with a marked reduction from 42.6 (U/L) to 22 (U/L) with a p-value of 0.04.

Table 3 illustrates the changes observed in 12 months in different laxative groups. TG and VLDL showed a significant change during the 12 months among the bulk-forming laxative group, with an increase from 1.72 (mmol/L) to 2.11 (mmol/L) and from 30.38 (mg/dL) to 37.38 (mg/dL), respectively, with a p-value of 0.04 for both. On the other hand, LDL levels appeared to decrease significantly in the combined group from 3.69 (mmol/L) to 2.65 (mmol/L) with a p-value of 0.04. Furthermore, cholesterol levels showed a marked and significant decrease among all laxative groups. This difference was significant between baseline and 12 months and when comparing laxative groups with a p-value <0.0001. A post hoc multivariant analysis was done on all parameters, and only the significant finding is presented in Table 4. The most noted finding was the changes in cholesterol during 12 months between the combined group and other groups, with the combined group showing the most decrease in cholesterol level, in addition to the differences seen between the osmotic and stimulant groups, with a p-value of 0.04.

**Table 3:** The 12-month change observed in BMI and different biochemical parameters in different laxative groups. TG, triglyceride; HDL, high-density lipoprotein; LDL, low-density lipoprotein; VLDL, very low-density lipoprotein; BMI, body mass index; HbA1c, hemoglobin A1c; ALT, alanine aminotransferase; AST, aspartate aminotransferase; ANOVA, analysis of variance. ^a^Paired test, ^b^one-way ANOVA, bold values with (*) are statistically significant (p-value < 0.05 ).

	n	Baseline	Month 12	Difference at month 12	p-Value	
					Baseline vs 12 months^a^	Between groups^b^
Cholesterol (mmol/L)						0.0001*
Osmotic	10	3.6 ± 0.4	4.4 ± 0.6	0.7 ± 0.3	0.03*	
Stimulant	9	3.9 ± 0.21	3.6 ± 0.17	-0.29 ± 0.2	0.24	
Bulk-forming	2	3.5 ± 0.35	4.6 ± 0.38	1.09 ± 0.005	0.003*	
Combined	6	5.08 ± 0.31	3.3 ± 0.31	-1.7 ± 0.3	0.008*	
TG (mmol/L)						0.12
Osmotic	10	1.1 ± 0.1	1.1 ± 0.1	0	0.98	
Stimulant	10	1.69 ± 0.28	1.59 ± 0.24	-0.09 ± 0.12	0.47	
Bulk-forming	2	1.72 ± 0.81	2.11 ± 0.78	0.4 ± 0.03	0.04*	
Combined	5	1.52 ± 0.28	1.05 ± 0.1	-0.47 ± 0.29	0.18	
HDL (mmol/L)						0.40
Osmotic	3	1.1 ± 0.2	1.2 ± 0.4	0.17 ± 0.14	0.35	
Stimulant	6	1.24 ± 0.16	1.23 ± 0.13	0.01 ± 0.08	0.95	
Bulk-forming	0	-	-	-	-	
Combined	1	1.18 ± 0	1.04 ± 0	-	-	
LDL (mmol/L)						0.06
Osmotic	6	2.8 ± 0.7	3.3 ± 0.7	0.48 ± 0.3	0.17	
Stimulant	9	2.22 ± 0.21	2.18 ± 0.17	-0.05 ± 0.28	0.87	
Bulk-forming	0	-	-	-	-	
Combined	3	3.69 ± 0.38	2.65 ± 0.43	-1.04 ± 0.23	0.04*	
VLDL (mmol/L)						0.12
Osmotic	10	20.1 ± 2.4	20.1 ± 2.3	0.07 ± 2.27	0.98	
Stimulant	10	29.87 ± 4.94	28.22 ± 4.22	-1.65 ± 2.17	0.47	
Bulk-forming	2	30.38 ± 14.3	37.38 ± 13.82	7 ± 0.44	0.04*	
Combined	5	26.96 ± 4.95	18.6 ± 1.84	-8.36 ± 5.22	0.18	
BMI (kg/m^2^)						0.65
Osmotic	20	28.2 ± 0.9	28.3 ± 1	0.17 ± 0.45	0.71	
Stimulant	26	30.83 ± 1.38	35.79 ± 5.49	4.95 ± 5.59	0.38	
Bulk-forming	11	29.07 ± 1.32	29.75 ± 1.36	0.68 ± 1.24	0.59	
Combined	17	26.49 ± 1.15	25.12 ± 1.01	-1.37 ± 0.61	0.04*	
HbA1c (%)						0.38
Osmotic	7	6.9 ± 0.8	6.1 ± 0.4	-0.86 ± 0.5	0.14	
Stimulant	7	8.48 ± 1.32	8.71 ± 1.29	0.23 ± 0.33	0.51	
Bulk-forming	4	7.62 ± 1.5	7.05 ± 1.04	-0.57 ± 0.74	0.50	
Combined	6	7.4 ± 0.9	6.85 ± 0.54	-0.55 ± 0.45	0.28	
ALT (U/L)						0.16
Osmotic	13	37.8 ± 9	24.1 ± 3	-13.69 ± 9.03	0.16	
Stimulant	13	21.38 ± 3.27	20.62 ± 3.73	-0.77 ± 3.32	0.82	
Bulk-forming	5	25.2 ± 2.76	33.4 ± 4.69	8.2 ± 3.87	0.10	
Combined	13	22.46 ± 2.36	17.08 ± 1.42	-5.38 ± 2.1	0.02*	
AST(U/L)						0.96
Osmotic	12	29.5 ± 5.1	29.8 ± 6.6	0.25 ± 8.98	0.98	
Stimulant	11	21.36 ± 2.17	23.73 ± 4.36	2.36 ± 4.15	0.58	
Bulk-forming	6	18.17 ± 3.48	24.67 ± 5.64	6.5 ± 6.01	0.33	
Combined	13	27 ± 7.57	32.46 ± 8.77	5.46 ± 10.72	0.62	

**Table 4:** Multivariant analysis of cholesterol change in 12 months. Bold values with (*) are statistically significant (p-value < 0.05 ) using post hoc (Tukey) multivariant analysis following the one-way ANOVA in Table 3. ANOVA, analysis of variance.

		Mean difference	Std. error	p-Value
Osmotic	Stimulant	1.06^*^	0.37	0.044
	Bulk-forming	-0.33	0.65	0.957
	Combined	2.47^*^	0.46	<0.0001*
Stimulant	Osmotic	-1.06^*^	0.37	0.044*
	Bulk-forming	-1.39	0.65	0.171
	Combined	1.41^*^	0.46	0.026*
Bulk-forming	Osmotic	0.33	0.65	0.957
	Stimulant	1.39	0.65	0.171
	Combined	2.80^*^	0.70	0.003*
Combined	Osmotic	-2.47^*^	0.46	<0.0001*
	Stimulant	-1.41^*^	0.46	0.026*
	Bulk-forming	-2.80^*^	0.70	0.003*

Interestingly, significant results were also noted on BMI levels in the combined group. A reduction in the mean BMI from 26.4 (kg/m^2^) to 25.1 (kg/m^2^) with a p-value of 0.04 was seen in this group. ALT levels also significantly decreased in the combined group from 22.46 (U/L) to 17.08 (U/L), with a p-value of 0.02. However, there was no significant change noted in the AST level.

To examine other factors that might affect these patients' lipid profiles, patients were categorized into two groups: patients who received statins (n = 54, 51%) and patients who did not receive statins (no statin) (n = 52, 49%).

The results were divided according to statin use for each parameter. The most significant finding in six months was seen in HbA1c, specifically in statin users, with a reduction in the mean HbA1c from 7% at baseline to 6.3% in the sixth month with a p-value of 0.002. HbA1c changes were also significantly different (p = 0.007) between the statin and no statin groups when analyzed using an independent t-test, indicating that the change might be due to statin use.

A significant change in cholesterol levels was also seen in both statin and no statin groups in 12-month periods compared to baseline. Cholesterol in the statin group decreased from 3.89 mmol/L to 1.62 mmol/L with a p-value of <0.0001 and dropped from 4.83 mmol/L to 2.18 mmol/L with a p-value of 0.003 in the non-statin group. Interestingly there was no statistical difference between the statin and non-statin groups with a p-value of <0.29.

Finally, ALT levels were markedly decreased in the no statin group in both periods, from 34.07 U/L to 23.52 U/L at six months with a p-value of 0.081 and from 31.87 U/L to 21.39 U/L at 12 months with a p-value of 0.065, with a nearly significant difference between statin and no statin groups with a p-value of 0.05. No other significance was noted in the other parameters (Table 5).

**Table 5:** The 12-month change observed in BMI and different biochemical parameters between statin and no statin groups TG, triglyceride; HDL, high-density lipoprotein; LDL, low-density lipoprotein; VLDL, very low-density lipoprotein; BMI, body mass index; HbA1c, hemoglobin A1c; ALT, alanine aminotransferase; AST, aspartate aminotransferase. ^a^Paired test, ^b^independent t-test,*p-value statistically significant.

	n	Baseline	Month 12	Difference at month 12	p-Value	
					Baseline vs 12 months^a^	Between statins vs no statins^b^
Cholesterol (mmol/L)						0.29
Statin	17	3.89 ± 0.2	1.62 ± 0.31	2.26 ± 0.31	<0.0001*	
No statin	10	4.83 ± 0.47	2.18 ± 0.54	2.65 ± 0.77	0.003*	
TG (mmol/L)						0.3
Statin	17	1.55 ± 0.18	1.52 ± 0.17	0.03 ± 0.12	0.822	
No statin	10	1.29 ± 0.21	1.1 ± 0.14	0.2 ± 0.13	0.166	
HDL (mmol/L)						0.77
Statin	8	1.24 ± 0.12	1.26 ± 0.12	-0.02 ± 0.08	0.779	
No statin	2	0.93 ± 0.32	1 ± 0.47	-0.08 ± 0.16	0.713	
LDL (mmol/L)						0.47
Statin	15	2.38 ± 0.27	2.27 ± 0.17	0.14 ± 0.24	0.671	
No statin	4	3.65 ± 0.78	3.87 ± 0.95	-0.22 ± 0.27	0.472	
VLDL (mg/dL)						0.38
Statin	17	27.4 ± 3.22	26.9 ± 3.03	0.5 ± 2.18	0.822	
No statin	10	22.9 ± 3.64	19.4 ± 2.41	3.51 ± 2.33	0.166	
BMI(kg/m^2^)						0.48
Statin	41	29.54 ± 1.02	29.86 ± 1.03	-.32 ± 0.43	0.885	
No statin	33	27.98 ± 0.73	31.12 ± 4.35	-3.13 ± 4.42	0.484	
HbA1c (%)						0.96
Statin	18	7.91 ± 0.69	7.5 ± 0.6	0.41 ± 0.31	0.202	
No statin	6	6.72 ± 0.41	6.28 ± 0.4	0.43 ± 0.34	0.256	
ALT(U/L)						0.05
Statin	21	21.62 ± 1.46	22.76 ± 2	-1.14 ± 1.78	0.527	
No statin	23	31.87 ± 5.54	21.39 ± 2.71	10.48 ± 5.39	0.065	
AST(U/L)						0.69
Statin	20	26.25 ± 4.98	27.75 ± 2.92	-1.5 ± 4.65	0.750	
No statin	22	28.77 ± 6.27	34.07 ± 5.88	-4.95 ± 7.13	0.494	

## Discussion

Laxatives are OTC products available without a prescription. The primary purpose of laxative administration is for constipation treatment. It has been reported that 75% of individuals with an average age between 40 and 80 years use laxatives [18]. Some studies also reported the use of laxatives for weight loss [4]. In the current study, we retrospectively investigated the possible effect of the chronic use of laxatives on the serum lipid profile and HbA1c.

Our results showed that chronic use of laxatives is more common in older people, with a mean age of 66 years in men and 63 years in females. Patients on chronic use of laxatives in our study showed comorbidities, including hypertension, heart failure, liver diseases, renal disorders, and cancer patients on chemotherapy. It has been reported that constipation is a common gastrointestinal (GI) disorder that increases with advancing age, particularly above 65 years of age, with an incidence range of 24% to 30% [19]. Hence, daily laxative use is expected, which matches our findings [20].

Furthermore, most cardiovascular diseases, including hypertension, heart failure, and chronic liver diseases, are associated with chronic constipation with regular laxatives intake by patients [21], as reported in Table 1 in the current study. In addition, constipation is prevalent in patients with chronic kidney disease (CKD), particularly in its advanced stages, due to their dietary limitations, associated comorbidities, concurrent medication use, and transformed gut microbiota [22]. Moreover, chemotherapy administration is usually associated with constipation. Therefore, cancer patients require laxatives for treating constipation and as a prophylactic measure [23].

In the current study, the most commonly used laxative type was stimulants, followed by the osmotic, while the bulk-forming was the least used. However, 25% of patients used a combination of more than one type. This is similar to a recent study investigating the types of laxatives used by patients, which showed that a significant category of patients use a combination of more than one type of laxative [24].

We studied the association between chronic laxative medications and different parameters, starting with the change in BMI. Our results showed that using combined laxatives for 12 months significantly reduced BMI levels. The association of weight loss with chronic laxative medication was reported by Levinson et al., 2020, who recommend not using laxatives as a weight control regimen due to its risk of developing eating disorders [25].

The combined group also showed a trend toward decreasing HbA1c within the 12-month users' group. This finding could explain the role of laxatives in regulating blood sugar. Laxatives regulate the amount of sugar absorbed, thus regulating blood glucose and HbA1c levels as previously described and explained by McRorie and McKeown (2017) and Gherbon et al. (2021) [26,27].

Despite the lowered HbA1c and, consequently, the glycemic state of the laxative user, it is still statistically nonsignificant. The use of laxatives by diabetic patients is expected due to diabetes-related chronic constipation [28], which is explained by diabetes-related dysmotility caused by the loss of enteric neurons and associated diabetic neuropathy [29]. Evidence-based therapeutic strategies for handling diabetes-related chronic constipation comprise lifestyle modification and laxatives. These laxatives include stimulants and osmotic agents [30]. Pieber et al. (2021) tested the effect of the chronic use of lactulose as an osmotic laxative for treating constipation in people with diabetes, which agrees with our findings [31]. Furthermore, Gherbon et al. (2021) reported a nonsignificant decrease in the blood glucose level, while fasting blood glucose level was found to be lowered [27]. These studies agree with mandatory continuous glycemic monitoring, especially in people with diabetes with chronic laxative intake.

Regarding the main objective of the current study, we investigated the impact of regular chronic laxative intake on plasma lipids profile. Our study revealed a significant decrease in plasma cholesterol and LDL-C levels in those who used laxatives for 12 months or more.

The cholesterol-lowering effect of laxatives has been previously reviewed by McRorie and McKeown 2016 [26]. Their review article reported that the bulk-forming laxative that depends on the presence of soluble fibers is associated with lowered total and LDL cholesterol levels. This finding is consistent with our results. The cholesterol-lowering effect of this type of laxative is explained by forming a fermentation byproduct of these fibers [32]. These fermentable fibers are considered prebiotics which can interfere with cholesterol absorption [26]. Trapping and eliminating bile via the stool is another possible mechanism. The excretion of bile provides an elimination method of cholesterol and decreases the digestion and absorption of dietary fat [33].

The reduced bile acid pool stimulates LDL receptor expression/increasing LDL cholesterol clearance to synthesize more bile acids. This clearance of LDL-C from the blood can efficiently lower serum total and LDL cholesterol without affecting HDL concentrations [33]. Another study by Asghar et al. (2018) investigated the effect of using laxative bael leaf extract on lipid profile. They revealed a lipid-lowering activity of bael leaf extract, which might be attributed to its phytoconstituents, such as saponins and phenolic ingredients, that help lower cholesterol levels (total and LDL-C) [34]. Stimulant laxatives increase water secretion into the large intestine lumen, promote intestinal motility, and increase the frequency of spontaneous bowel movements [35,36]. Some stimulant laxatives, such as elobixibat, bind and hinder bile acid transporters in the ileal mucosa, thereby enhancing bile acid excretion. As mentioned above, this effect will stimulate the production of bile acids from cholesterol in the liver [37] and subsequently reduce serum cholesterol concentration [8].

Regarding osmotic laxatives, it was found that alteration of the intestinal microbiome composition inhibits the metabolism of the primary bile salts, enhancing its excretion and modifying cholesterol metabolism, which delays its absorption [9]. Their results were also confirmed later by Strisciuglio et al.'s (2021) study [38]. Most patients with chronic constipation, especially the geriatric age group and those with associated chronic diseases (hypertension, heart failure, liver disease, chronic kidney disease, and cancer), use a combination of more than one type of laxative simultaneously [39]. Thus, this can induce multiple mechanisms mediating the cholesterol-lowering effect. This is consistent with our finding in which a significant impact was noted in this patient group.

The current study showed a significant increase at 12 months in TG and VLDL levels in those who used bulk-forming laxatives. VLDL and TG levels depend on each other regarding their plasma level. VLDL is a TG-rich particle responsible for the hepatic export of endogenous TGs to the extrahepatic tissue [40]. The close link between constipation could explain the increased TG (total and VLDL-TG) plasma level as the actual problem of those patients and dyslipidemia in the form of an abnormal increase in TG level [41,42]. One possible explanation for this link is that constipation can lead to increased absorption of dietary fat. When food passes through the colon slowly, the body has more time to absorb the fat [43]. This can lead to increased TG levels in the blood. Another possible explanation is that constipation can lead to inflammation in the gut. Inflammation can damage the cells that line the gut, making it easier for TGs to leak into the bloodstream [44].

Additionally, there was a significant decrease in plasma ALT activity in the combined laxative group after 12 months, but still within the normal reference range. However, no change in AST levels was observed. This is similar to Lee et al.'s (2020) study, which showed the possible effect of laxative use on ALT levels [45]. The laxative use can explain this associated lipid-lowering impact, which decreases the possibility of hepatic lipid accumulation, thus preventing hepatic stress. 

To prove that the observed changes in total cholesterol and LDL-C are due to chronic laxative use or the associated lipid-lowering drug intake, the current study investigating the effect of statin (the cholesterol-lowering drug) on BMI and HbA1c showed no significant difference after 12 months of chronic intake of laxatives compared to baseline. The same finding was observed when comparing cholesterol levels after 12 months of laxative intake. As mentioned above, the plasma cholesterol level significantly decreased after 12 months from the baseline levels in both groups (statin-treated and non-statin-treated groups). This difference in the current study might not be solely because of statin since there was no statistical significance between the statin-treated and non-statin-treated groups, as the p-value of the independent T-test is insignificant. However, statin appears to have a synergistic effect as the statin group has a more significant change. However, Derosa et al. (2015) reported no synergistic effect of laxatives and statins in lowering blood cholesterol. There is no evidence that taking laxatives and statins will lower blood cholesterol any more than taking statins alone in their study [46]. So, we can recommend the strict follow-up of the serum lipids and glycemic parameters in those patients with chronic laxative intake. 

Limitations of this study include the small sample size originating from a single center. Not all patients had all their laboratory parameters at all time points (baseline, six, or 12 months). Furthermore, some patients' daily doses of laxatives were not fully described. Moreover, statin dose, duration, and compliance were not recorded. In addition, the dietary habits of individuals were also not recorded.

## Conclusions

This retrospective study concluded that chronic laxative use is associated with lowered glycemic parameters (HbA1c) and reduced body weight (BMI). Also, using laxatives is associated with a decreased plasma level of total and LDL cholesterol with no significant effect on HDL-C levels. Surprisingly, total and VLDL TG plasma levels increased. Moreover, chronic use of laxatives is associated with lowered hepatic function tests, which may be secondary to its cholesterol-lowering effect with statin co-treatment as a confounding factor.

Finally, continuous monitoring of the glycemic and lipid parameters, as well as LFT, is recommended in all patients receiving laxatives for six months or more. Moreover, physical activity and statin dosing also should be considered. Further multicentric studies on larger sample sizes are advised to confirm our findings.
